# A pathogenic *AKAP4* variant, p.R429H, causes male in/subfertility in humans and mice

**DOI:** 10.1002/ctm2.1463

**Published:** 2023-12-04

**Authors:** Han Wei, Xiaohui Zhang, Chunyan Wang, Jing Wang, Tengyan Li, Suren Chen, Hongjun Li, Binbin Wang

**Affiliations:** ^1^ Center for Genetics National Research Institute for Family Planning Beijing China; ^2^ Graduate School Chinese Academy of Medical Sciences & Peking Union Medical College Beijing China; ^3^ Center for Reproductive and Genetic Medicine Dalian Municipal Women and Children's Medical Center Dalian China; ^4^ Key Laboratory of Cell Proliferation and Regulation Biology Ministry of Education Department of Biology College of Life Sciences Beijing Normal University Beijing China; ^5^ Department of Medical Genetics and Developmental Biology School of Basic Medical Sciences Capital Medical University Beijing China; ^6^ Department of Urology Peking Union Medical College Hospital Beijing China; ^7^ NHC Key Laboratory of Reproductive Health Engineering Technology Research (NRIFP) Beijing China

Dear Editor,

Azoospermia, oligozoospermia and asthenozoospermia are well‐established causes of male infertility. Next‐generation sequencing has contributed to understanding Mendelian forms of male sterility.^1^ We identified a pathogenic hemizygous *AKAP4* variant (c.1286G > A/p.R429H) shared by two siblings suffering from non‐obstructive azoospermia (NOA), while a different missense change involving the same amino acid residue, p.R429C, caused multiple morphological abnormalities of the sperm flagellum (MMAF) and severe oligozoospermia in a prior study.^2^ An equivalent *Akap4* ^R428H^ mutation knock‐in mouse model was generated using CRISPR/Cas9 technology, which exhibited pronounced male subfertility characterized by diminished sperm count and motility, as well as fibrous sheath (FS) abnormalities in the flagella.

**FIGURE 1:**
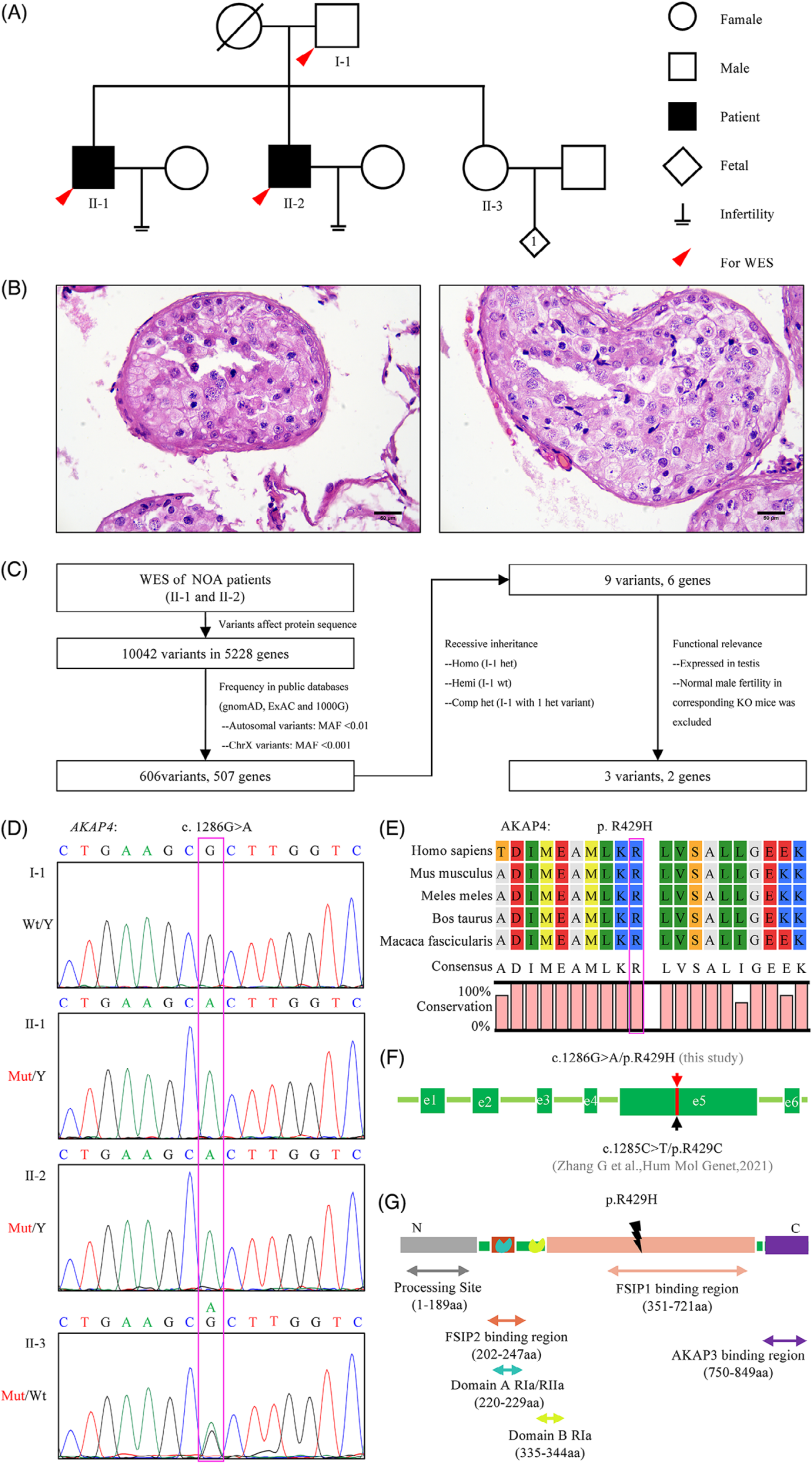
Pedigree of the NOA family and identification of candidate variants by whole‐exome sequencing. (A) Pedigree of the non‐obstructive azoospermia family which included two probands. WES, whole‐exome sequencing. (B) Representative images of testicular tissue stained with hematoxylin‐eosin from proband II‐1 showed comprehensive meiotic arrest. Scale bars, 50 μm. (C) Flow chart of bioinformatics analysis of whole exome sequencing data. NOA, non‐obstructive azoospermia; KO, knockout. (D) Sanger sequencing of pedigree members to validate the *AKAP4* candidate variant genotype. (E) Conservation analysis of the *AKAP4* R429 amino acid. (F) *AKAP4* candidate mutation located in exon 5 of the *AKAP4* gene. (G) The missense mutation of AKAP4 p.R429H located in the *AKAP4* protein FISP1 binding region.

A‐kinase anchor protein 4 (*AKAP4*), an X chromosome‐linked gene, is exclusively expressed in spermatids and mature spermatozoa in previous studies.^3^ AKAP4 participates in tethering Cyclic‐AMP dependent protein kinase A (PKA) to substrates for protein phosphorylation and constructing FS skeleton structure.^4^ Recently, testicular single‐cell transcriptomic studies^5^, ^6^ have indicated that *AKAP4*, which escapes meiotic sex chromosome inactivation, is also expressed in spermatogonia and spermatocytes, despite lower expression than in spermatids. This knowledge suggests that AKAP4 might function beyond flagella development during spermatogenesis. Notably, variants in the *AKAP4* gene have been identified in infertile males exhibiting distinct phenotypes, such as asthenozoospermia,^7^ MMAF,^2^ and azoospermia/NOA,^8^, ^9^ which shed light on the divergence of male infertility phenotypes of *AKAP4* mutations.

We recruited a family including two brothers with histopathologically confirmed NOA (Figure 1A,B). The characteristics of the two probands (II‐1, II‐2) are shown in Table 1. After precluding common etiological factors, whole‐exome sequencing was applied to search for genetic causes (Figure 1C). After strictly filtering, six genes remained: three showed biallelic missense mutations (*OBSCN*, *SYNE1* and *ZNF282*), and three had X‐linked missense mutations (*AKAP4*, *PLXNB3*, and *SRPK3*) (Table S1, Figure S1). The contributions of mutations in *OBSCN*, *SYNE1*, *PLXNB3* and *SRPK3* to NOA phenomena were excluded by previous knockout (KO) mouse studies through a search of the Mouse Genome Informatics database (https://www.informatics.jax.org/). Segregation analysis was applied to identify hemizygous *AKAP4* variation (Figure 1D, Table S2) and compound heterozygous *ZNF282* variations (Figure S2). *AKAP4* c.1286G > A/p.R429H is located in exon 5, and in silico information is detailed in Table 1 and Figure 1E–G. As yet, there is no literature report of *Akap4* homologous mutant knock‐in and *Zfp282*‐KO mouse models (mouse ZFP282 is orthologous to human ZNF282), so we constructed corresponding mice to explore the effect of these variants on male fertility.

**TABLE 1:** Clinical information of the two non‐obstructive azoospermia (NOA) siblings and the in silico information of the hemizygous deleterious *AKAP4* variant identified in the two probands.

	II‐1	II‐2
Age	35 years	28 years
Type of sterility	Primary	
Medical history	None	
Physical examination	Normal	
Semen analysis	No sperm	
Testicular biopsies	Maturation arrest	
Karotype	46, XY	
Y micro‐deletion detection	Normal	
Hormone levels		
E2 (20–47)	28.00 pg/mL	25.99 pg/mL
FSH (1.3–19.3)	3.76 mIU/mL	5.52 mIU/mL
LH (1.2–8.6)	6.96 mIU/mL	3.46 mIU/mL
PRL (2.3–13.1)	7.62 ng/mL	9.78 ng/mL
T (1.75–7.81)	6.53 ng/mL	4.73 ng/mL
*AKAP4* variant		
cDNA alteration	c.1286G > A	
Variant allele	Hemizygous	
Protein alternation	p.R429H	
Variant type	Missense	
Allele frequency in human population		
All individuals in gnomAD	.0001	
East Asians in gnomAD	0	
1000 Genomes Projects	0	
Function prediction		
SIFT	Damaging	
PolyPhen‐2	Damaging	
MutationTaster	Disease_causing	
CADD	24.3	
Conservation analysis		
GERP++	Conserved	
PhyloP	Conserved	
SiPhy	Conserved	

*Note*: NCBI reference sequence number of *AKAP4* is NM_003886. Variants with CADD value >15 are considered to be deleterious.

*Zfp282*‐KO mice (Figure S3A–C, Table S3 and S4) were viable and exhibited no overt abnormalities. A series of experiments were applied to test the fertility of *Zfp282*‐KO mice, including fertility test (Figure S3D), sperm counts and motility (Figure S3E), H&E staining of testis/epididymis sections and Papanicolaou staining of sperm (Figure S3F), which collectively showed that there is no impact of ZNF282 on male fertility. We generated *Akap4* p.R428H mice (Figure 2A,B, Table S5 and S6), which is equivalent to the human *AKAP4* ^R429H^ mutation. The AKAP4 protein was significantly reduced in the testes of *Akap4* ^R428H^ mice (Figure 2C), indicating that the R428H variant might affect AKAP4 protein stability. The fertility test indicated severe male subfertility in which only 8/24 female mice mated with *Akap4* ^R428H^ males were pregnant and produced 37 offspring; in contrast, 24/25 female mice mated with wild‐type (WT) males became pregnant and gave rise to 203 offspring (Figure 2D). The male reproductive system, testis/body weight ratio and histological examination of testis sections of *Akap4* ^R428H^ mice were not obviously different from those of WT mice (Figure 2E–F). Transmission electron microscopy analysis showed no obvious abnormalities in manchette structure of spermatids in *Akap4* ^R428H^ mice (Figure S4).

**FIGURE 2:**
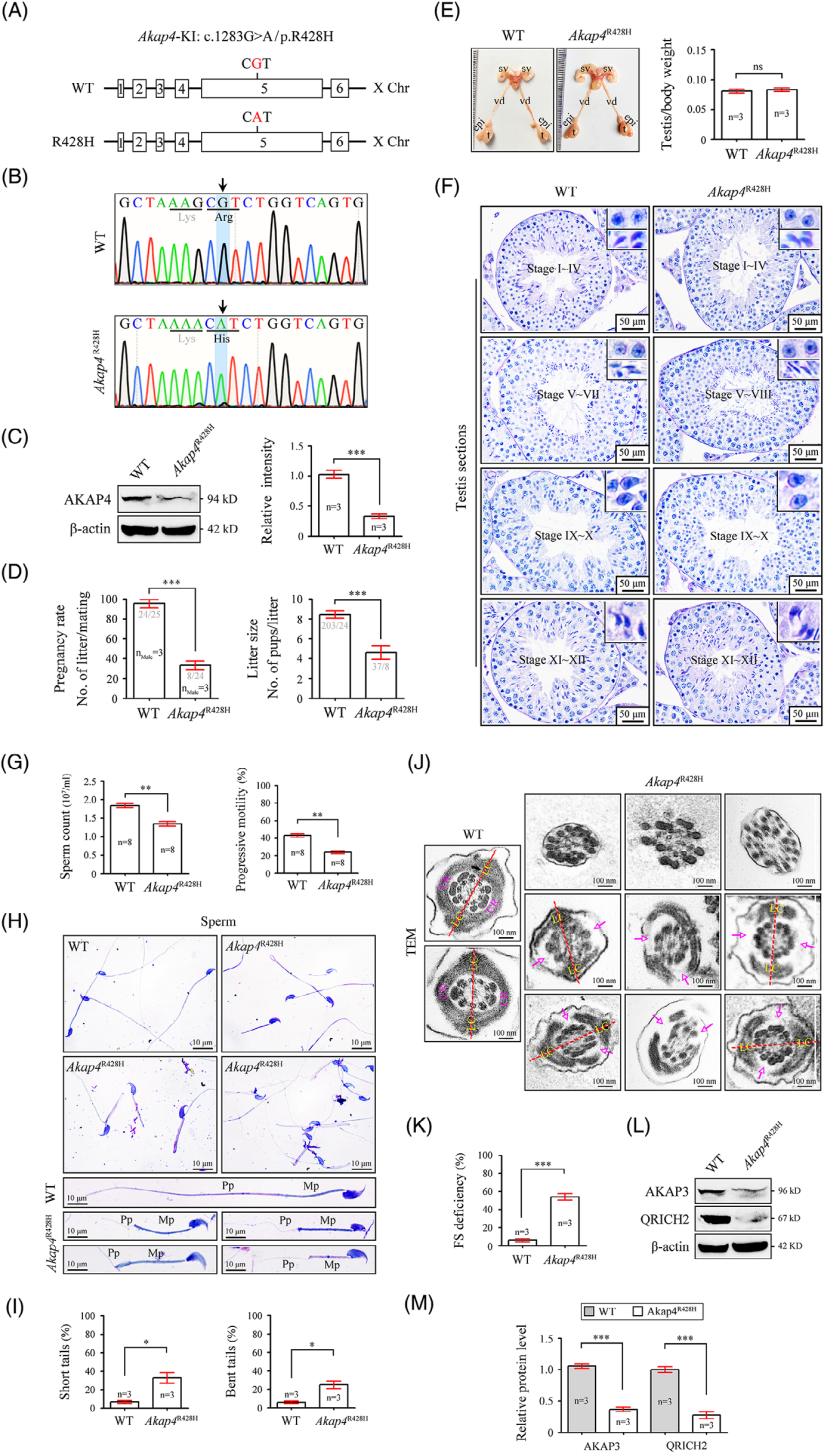
The equivalent mutation in the mouse Akap4 gene leads to male subfertility and the *Akap4* ^R428H^ mice show reduced sperm count/motility and a defective fibrous sheath. (A) Schematic illustration of the targeting strategy for generating *Akap4* ^R428H^ (CGT to CAT) mice by using CRISPR/Cas9 technology. (B) Representative results of Sanger sequencing‐based genotyping using tail DNA. (C) Immunoblotting of *AKAP4* was performed in wild‐type (WT) and *Akap4* ^R428H^ testes. β‐actin served as a loading control. Bar graphs represent band intensities of blots, and data represent the mean ± SEM of three biological replicates. Student's t‐test, ***p 〈 .001. (D) A fertility test was performed in three *Akap4* ^R428H^ male mice and three littermate WT male mice for 2 months. Each male mouse was mated with two C57BL/6J female mice. Vaginal plugs were observed in all mated females, and the number of pups per litter was recorded. Data are presented as the mean ± SEM. Student's t test, ***p 〈 .001. (E) The morphology of the male reproductive system and the testis/body weight ratio of Akap4R428H mice and WT mice. t, testis; epi, epididymis; vd, vas deferens; sv, seminal vesicle. Data represent the mean ± SEM of three biological replicates. Student's t test, ns, not significant. (F) Periodic acid‐Schiff staining of testis sections from Akap4R428H mice and WT mice. According to the components of the spermatids, the seminiferous tubules can be divided into stages I–XII. The differentiating spermatids within each stage are enlarged and shown in the upper right corners of the figures. Scale bars, 50 μm. (G) Sperm from the cauda epididymis of *Akap4* ^R428H^ mice and WT mice. The sperm number was counted with a fertility counting chamber under a light microscope, and sperm motility was assessed by a computer‐assisted sperm analysis (CASA) system. Data are presented as the mean ± SEM (n = 8 each group). Student's t test was performed, **p 〈 .001. (H) Morphological analyses of epididymal sperm in *Akap4* ^R428H^ mice and WT mice by Papanicolaou staining. Mp, mid‐piece; Pp, principal piece. Scale bars, 10 μm. (I) Percentage of sperm with short tails or bent tails from the cauda epididymis of Akap4R428H mice and WT mice. At least 100 sperm were counted for each mouse. Data are presented as the mean ± SEM (n = 3 each group). Student's t test was performed, *p 〈 .05. (J) Epididymal sperm from WT mice and *Akap4* ^R428H^ mice were fixed and sectioned for transmission electron microscopy. Cross‐sections of the principal piece of flagella are shown. The vacant positions of the fibrous sheath (FS) are indicated by arrows. LC, longitudinal column; CR, circumferential ribs. Scale bars, 100 nm. (K) Percentage of sperm with FS deficiency in *Akap4* ^R428H^ mice and WT mice. At least 50 principal pieces of flagella were counted for each mouse. Data are presented as the mean ± SEM (n = 3 each group). Student's t test was performed, *p 〈 .05, ***p 〈 .001. (L and M) Immunoblotting of *AKAP3* and QRICH2 was performed in WT and *Akap4* ^R428H^ testes. β‐actin served as a loading control. Bar graphs represent band intensities of blots. Mean ± SEM of three biological replicates. Student's t test, ***p 〈 .001.

To illustrate the cause of reduced fertility in *Akap4* ^R428H^ mice, we performed sperm analysis using mature sperm from the cauda epididymis. Significantly reduced sperm count and progressive motility were identified in *Akap4* ^R428H^ mice (Figure 2G). Papanicolaou staining further indicated that *Akap4* ^R428H^ mice produced short‐tailed and bent‐tailed sperm with dramatically attenuated principal piece (Figure 2H). Short‐tailed and bent‐tailed sperm accounted for approximately 33% and 25% of the total sperm in *Akap4* ^R428H^, respectively (Figure 2I). TEM showed that FS was either unrecognizable or partially lost (Figure 2J). The ratio of FS deficiency in sperm from *Akap4* ^R428H^ was significantly higher than that in WT sperm (54.00% vs. 5.90%) (Figure 2K). We further found that the protein expression of AKAP3 (a FS protein) and QRICH2 (a known target of AKAP4) was significantly lower in the testis lysates of *Akap4* ^R428H^ mice (Figure 2L,M). Collectively, these data confirmed that the R429H variant of *AKAP4* is a pathogenic mutation to cause male in/subfertility.

To exploit the regulatory mechanism of AKAP4 in mouse spermatogenesis, we reanalyzed the single‐cell transcriptome data of *Akap4*‐KO and WT testes from the Sequence Read Archive database (access number: SRR9107534),^10^ which were detailed in the supplementary Material and Methods (Figure S5A,B). Through differential expression analysis without distinguishing cell types we identified thirteen main differentially expressed genes (DEGs) (|log2FC|≥.5, adjusted *p* < .05) including *Ccdc38* and *Haspin*, which were reported in previous literature^10^ (Figure S5C). Trajectory analysis provided novel findings compared with the previous results.^10^ The shorter velocity vectors indicated a decreased accumulation of mRNA in *Akap4*‐KO mice (Figure S6A). Round spermatids (RSs) were positioned at the starting point of the pseudotime trajectory in KO mice, whereas spermatocytes served as the early state of cell differentiation in WT mice (Figure S6B,C). Enrichment of RS DEGs did not show a significant pathway for spermatogenesis (Figure S7A–C). Protein‐protein interaction network (PPI) analysis of these DEGs showed potential AKAP4 interacting partners (Figure S7D,E). Both up/downregulated DEGs in elongating spermatids were significantly enriched in spermatogenesis (Figure S6D,E), and PPI revealed a central node containing AKAP4, H1FNT (Figure S6F,G), suggesting that AKAP4 may act through its interactors to mainly affect the late stages of spermiogenesis.

In conclusion, the functional alterations in AKAP4 have been demonstrated to exert significant contributions to male infertility, encompassing asthenozoospermia (including MMAF), severe oligozoospermia and even a complete failure of spermatogenesis (Figure 3). The underlying mechanisms of AKAP4 mutations leading to NOA and the phenotypic difference between the *AKAP4* p.R429H variant in humans and *Akap4* ^R428H^ mice remain to be studied in the future.

**FIGURE 3:**
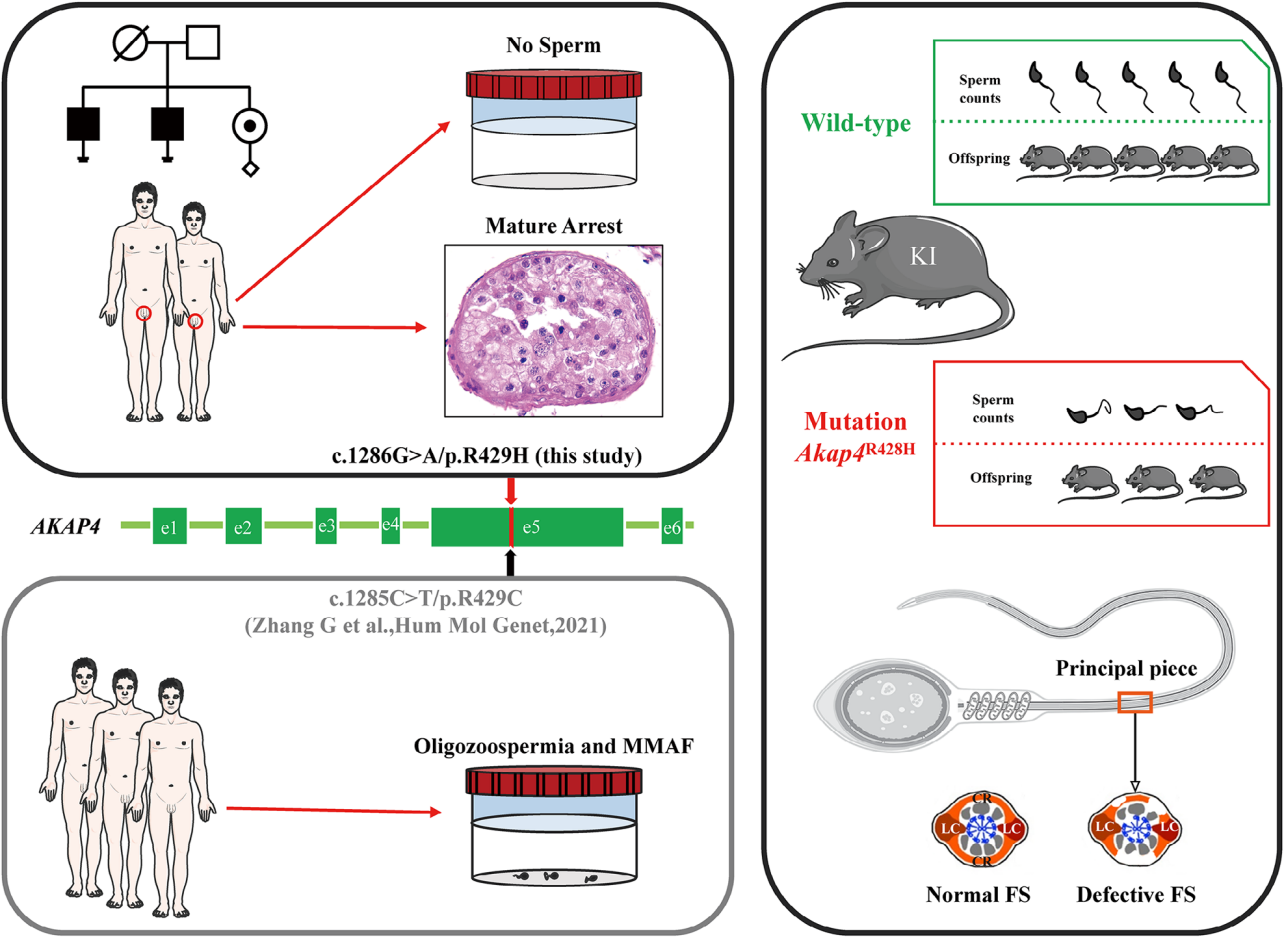
A pathogenic hemizygous variant in *AKAP4* causes azoospermia in humans and severe male subfertility in mice. A pathogenic hemizygous *AKAP4* variant (c.1286G〉A/p.R429H) was identified in two siblings suffering from non‐obstructive azoospermia, while a different missense change involving the same amino acid residue, p.R429C, has been associated with multiple morphological abnormalities of the sperm flagellum (MMAF) and severe oligozoospermia in a prior study. To further validate the pathogenicity of this mutation on *AKAP4* function and male fertility, we constructed corresponding mutant (knock‐in) mice using CRISPR/Cas9 technology. *Akap4* ^R428H^ mice displayed severe male subfertility, showing reduced sperm count/motility and fibrous sheath abnormalities in flagella. Together, our study provides clinical and animal evidence to reveal a hemizygous variant in *AKAP4* (c.1286G〉A/p.R429H) causes male in/subfertility in both humans and mice.

## AUTHOR CONTRIBUTIONS

Wei H and Zhang XH performed the major experiments and wrote the manuscript. Wang CY. and Wang J undertook the bioinformatics analysis. Li TY completed Sanger sequencing. Chen SR, Li HJ and Wang BB designed the study and revised the manuscript. All authors approved the final version for submission.

## CONFLICT OF INTEREST STATEMENT

The authors declare no competing interests in relation to publication of this study.

## FUNDING INFORMATION

This work was supported by the Beijing Municipal Natural Science Foundation (7232112), the National Key Research and Development Project (2019YFA0802101) and the Open Fund of Key Laboratory of Cell Proliferation and Regulation Biology, Ministry of Education.

## ETHICS STATEMENT

This study was approved by the ethics committee from the Peking Union Medical College Hospital and National Research Institute for Family Planning. Animal experiments were approved by the Animal Care and Use Committee of the College of Life Sciences, Beijing Normal University.

## Supporting information

Supporting information

## Data Availability

The datasets generated during the current study are available from the corresponding author upon reasonable request.
