# Supplementary material for: A pathogenic AKAP4 variant, p.R429H, causes male in/subfertility in humans and mice
Source: Clin Transl Med. 2023 Dec 4;13(12):e1463. doi: 10.1002/ctm2.1463 (PMC10696161; doi:10.1002/ctm2.1463)

**A pathogenic AKAP4 variant, p.R429H, causes male in/subfertility in humans and mice**

**Running title:** LOF mutation in *AKAP4* causes male in/subfertility

Han Wei^1,2,3*^, Xiaohui Zhang^4*^, Chunyan Wang^1,2^，Jing Wang^5^, Tengyan Li^1^, Suren Chen^4^, Hongjun Li^2,6^, Binbin Wang^1,2^

1 Center for Genetics, National Research Institute for Family Planning, Beijing, 100000, China.

2 Graduate School, Chinese Academy of Medical Sciences & Peking Union Medical College, Beijing, 100000, China.

3 Center for Reproductive and Genetic Medicine, Dalian Municipal Women and Children's Medical Center, Dalian, 116000, China.

4 Key Laboratory of Cell Proliferation and Regulation Biology, Ministry of Education, Department of Biology, College of Life Sciences, Beijing Normal University, Beijing, 100000, China.

5 Department of Medical Genetics and Developmental Biology, School of Basic Medical Sciences, Capital Medical University, Beijing, 100000, China.

6 Department of Urology, Peking Union Medical College Hospital, Beijing, 100000, China.

* Han Wei and Xiaohui Zhang contributed equally to this work.

**Correspondence:**

Binbin Wang, E-mail: wbbahu@163.com. ORCID: https://orcid.org/0000-0002-1597-5930.

Hongjun Li, E-mail: lihongjun@pumch.cn.

Suren Chen, E-mail: chensr@bnu.edu.cn.

**Supplementary Materials and Methods**

**Patients**

A nonconsanguineous family exhibiting non-obstructive azoospermia (NOA) was recruited in the Andrology clinic and included two siblings diagnosed with NOA as the probands, their fertile father, and an unaffected younger sister. NOA was diagnosed by consecutive semen analysis and histological analysis of testicular biopsies. G-banding karyotype and Y-chromosome AZF microdeletion analysis were routinely performed. Other risk factors were excluded, including cryptorchidism, radiotherapy and chemotherapy, viral or bacterial orchitis, epididymitis, epididymo-orchitis, undescended testis, hypogonadism, and sexually transmitted infections. Peripheral blood was collected from the family for next-generation and Sanger sequencing. Informed consent was obtained from all subjects.

**Whole exome sequencing**

Whole-exome sequencing was performed using genomic DNA extracted from blood collected from both affected members and their fertile father. Briefly, all exons were captured by using SureSelect Human All Exon V6 Enrichment kits (Agilent, Santa Clara, CA, USA) and then sequenced on a NovaSeq platform (Illumina, San Diego, CA, USA). Reads were mapped to the human reference genome (GRCh37) by Burrows‒Wheeler Alignment v0.7.9a^1^ (http://bio-bwa.sourceforge.net). Single nucleotide variants and indels were called by Genome Analysis Toolkit v3.5^2^ (https://gatk.broadinstitute.org) and annotated through ANNOVAR software^3^ (https://annovar.openbioinformatics.org).

**Variant filtration**

To find candidate pathogenic variants, filtering criteria were used as follows: (1) missense, nonsense, frameshift or canonical splicing variants; (2) autosomal chromosome variants with a minor allele frequency (MAF) less than 1%, or X chromosome hemizygous variants with a MAF less than 0.1% in the Genome Aggregation Database (gnomAD, http://gnomad.broadinstitute.org); (3) both affected members shared two compound heterozygous variants with only one from the father, a homozygous variant, or a hemizygous variant that the father did not have; and (4) variants were in silico predicted as deleterious by at least two of the Sorting Intolerant From Tolerant (SIFT)^4^, PolyPhen2^5^ and MutationTastor^6^ programs. Sequence conservation analysis was applied by CLC Sequence Viewer 8 software.

**Candidate genes expression in the testis**

The online testicular single-cell transcriptome databases in The Human Protein Atlas (https://www.proteinatlas.org/) and Single Cell Expression Atlas (https://www.ebi.ac.uk/gxa/sc/) were used to query the expression levels of candidate genes in different cells of the testis. Murine testicular gene mRNA expression levels were shown by uniform manifold approximation and projection diagram (parameters including k = 21 and n_neighbors:100) from the Single Cell Expression Atlas online website and data were derived from the mouse testis single-cell transcriptome dataset by Ernst et al^7^. The germ cell population was annotated with cardinal marker genes used by Ernst and colleagues, including spermatogonia (*Dmrt1*), spermatocytes (*Piwil1*), round spermatids (*Tex21*) and elongating spermatids (*Tnp1*).

**Variant validation**

PCR was performed with LongAmp Taq 2X Master Mix (Takara, Otsu, Shiga, Japan) using customized primers (BGI, Inc.) (Table S2). After amplification confirmation with agarose gel electrophoresis and purification with a QIAquick PCR Purification Kit (QIAGEN), Sanger sequencing reactions of PCR products were carried out with an ABI3730xl Genetic analyzer. Sequence analysis and visualization of the chromatograms was performed with Codon Code Aligner software (v.8.0.1).

**Mouse model**

The mouse *Akap4* gene (GenBank accession number: NM_009651.4; Ensemble: ENSMUSG00000050089) is located in mouse chromosome X and contains six exons, with the ATG start codon in exon 1 and TAA stop codon in exon 6. The codon encoding R428H is located in exon 5. The gRNA and donor oligo were designed and synthesized by Sangon Biotech (Shanghai, China). Cas9 mRNA, gRNA, and donor oligo were coinjected into C57BL/6JGpt fertilized eggs. The injected zygotes were transferred into pseudopregnant recipients to obtain F0 generation offspring. The pups were genotyped by PCR using a Mouse Tissue Direct PCR Kit (YEASEN, 10185ES50) followed by sequencing analysis. The targeting strategy, gRNA target sequence, donor oligo sequence, and primer sequence for genotyping are provided in Table S5.

The mouse Zfp282 is orthologous to human ZNF282. *Zfp282* gene (MGI: 2141413, Ensemble: ENSMUSG00000025821) is located in mouse chromosome 6 with genomic length of 31,282 bp. *Zfp282*-KO mice were generated to assess its physiological role in spermatogenesis. A 13,824 bp fragment containing exons 2~5 of the *Zfp282* gene was selected for deletion by using CRISPR/Cas9 technology (Fig. S3A, Table S4).

**Reverse transcription PCR (RT‒PCR)**

Total RNA was extracted from the testes of Zfp282-KO mice and their littermate WT mice using an RNA Easy Fast Tissue/Cell Kit (Tiangen Biotech). Approximately 0.3 mg total RNA was converted into cDNA with a FastKing One-Step RT‒PCR Kit (Tiangen Biotech) according to the manufacturer’s instructions. The cDNAs were used as templates for the subsequent RT‒PCR with PCR mix (Tiangen Biotech). Mouse *Gapdh* was used as an internal control. Primers for RT‒PCR are listed in Table S3.

**Fertility testing**

The fertility of *Akap4*^R428H^ mice was examined by natural mating tests. Briefly, three Akap4^R428H^ and three littermate control and sexually mature male mice (8 to 12 weeks old) were paired with two 6–8-week-old C57BL/6J females (male:female ratio of 1:2) for 2 months. Mice were examined for vaginal plugs every morning. Female mice with vaginal plugs were separately fed and the number of pups per litter was recorded.

**Histological analysis**

Testis tissues from adult mice were dissected and fixed in Bouin’s solution (Bedebio, HD15990) for 4 h at 4°C. Fixed tissues were embedded in paraffin, sectioned (5 μm thick), dewaxed and rehydrated. The sections were stained with Periodic Acid Schiff’s solution (Solarbio, G1280) before imaging using a Leica DM-500 optical microscope (Leica Microsystems, German).

**Assessment of sperm counts and motility**

The backflushing method was used to retrieve sperm from the cauda epididymis^8^. Sperm counts were determined using a Fertility Counting Chamber (Makler, Israel) under a light microscope and sperm mobility was assessed via the application of a computer-assisted sperm analysis system (Hamilton Thorne-TOX IVOS, USA).

**Sperm morphology**

Sperm were collected from the cauda epididymis and washed three times in PBS buffer. The sperm suspension was mounted on a glass slide, air-dried, and fixed with 4% PFA for 10 min at room temperature. The slides were stained with Papanicolaou solution (Solarbio, G2571) and observed using a Leica DM-500 optical microscope (Leica Microsystems, German).

**Transmission electron microscopy**

Precipitated mouse sperm (~1 mm3) were fixed with 2.5% (vol/vol) glutaraldehyde in 0.1 M phosphate buffer (PB) (pH 7.4) for 24 h at 4°C. Samples were washed four times in PB and first immersed in 1% (wt/vol) OsO4 and 1.5% (wt/vol) potassium ferricyanide aqueous solution at 4°C for 2 h. After washing, the samples were dehydrated through graded alcohol solutions (30%, 50%, 70%, 80%, 90%, 100%, and 100%, 10 min each) into pure acetone (10 min, two times). Samples were infiltrated in graded mixtures (3:1, 1:1, and 1:3) of acetone and SPI-PON812 resin (21 ml SPO-PON812, 13 ml dodecenylsuccinic anhydride, and 11 ml nadic methyl anhydride) and then pure resin. The specimens were embedded in pure resin with 1.5% benzyldimethylamine, polymerized for 12 h at 45°C and 48 h at 60°C, cut into ultrathin sections (70 nm thick), and then stained with uranyl acetate and lead citrate for subsequent observation and photography with a Tecnai G2 Spirit 120 kV (FEI, Lausanne, Netherlands) electron microscope. All reagents were purchased from Zhongjingkeyi Technology (Beijing, China).

**Western blot**

Testicular proteins were extracted using RIPA lysis buffer (Applygen, C1053) containing 1 mM phenylmethylsulfonyl fluoride and protease inhibitors on ice. The supernatants were collected following 12,000 g centrifugation for 20 min. Proteins were electrophoresed in 10% SDS‒PAGE gels and transferred to nitrocellulose membranes (GE Healthcare, WI, US). The blots were blocked in 5% milk and incubated with primary antibodies overnight at 4°C, followed by incubation with anti-rabbit or mouse IgG(H&L)-HRP (Abmart, M212108, M212115) at a 1/10,000 dilution for 1 h. The detection of signals was evaluated using Super ECL Plus Western Blotting Substrate (Applygen, P1050) and a chemiluminescence imaging system (Tanon, Shanghai, China). The following primary antibodies were used in this study: rabbit polyclonal to AKAP3 (Proteintech, 13907-1-AP), mouse monoclonal to AKAP4 (Santa Cruz, sc-135827), mouse monoclonal to QRICH2 (Santa Cruz, sc-514279), and mouse monoclonal to β-actin (Abcam, ab8226).

**Single-cell RNA sequencing data analysis**

Raw single-cell RNA-seq data of testis single cells from *Akpa4*-KO mice and wild-type control mice were downloaded through NCBI online database (accession number: SRR9107534). Cellranger software (version 5.0.1) was applied for quality control and data filtering using filtering criteria including minimum number of expressed genes per cell (20), doublets, minimum number of cells per gene (200), mitochondrial proportions (15%), and cell cycle correction. We obtained a total of 4998 and 5107 testicular cells from *Akpa4*-KO and WT mice, respectively, for downstream analysis. The RNA reads were aligned to the mm10-2020-A reference genome using STAR software. Downstream analyses were performed using Seurat software (version 3.0.2) for dimensionality reduction and clustering and SCSA software for cell annotation. Testicular single-cell annotations were based on marker genes, including spermatogonia (*Stra8*, *Hormad1*, *Sycp1*), spermatocytes (*Tbpl1*, *Piwil1*, *Spo11*), round spermatids (*Tssk1*, *Acrv1*, *Spaca1*), elongating spermatids (*Prm1*, *Prm2*, *Tnp1*, *Tnp2*), Sertoli cells (*Amhr2*, *Sox9*, *Clu*, *Ctsl*), macrophages (*Cd81*, *Cd74*), and other somatic cells. Differential expression genes (DEGs) were called by by using FindMarkers (Seurat 3.0.2) from the same cluster between different samples with filtering criteria of |log2FC|≥0.5 and adjusted p<0.05. Enrichment was made on four databases: Gene ontology (Cellular Component, Molecular Function and Biological Process) and KEGG as described before^9^. Velocyto software (version: 0.17.17) was used to apply single-cell RNA velocity analysis. RNA velocity is a high-dimensional vector that predicts future cell states by measuring the time-dependent relationship between precursor and mature mRNA levels^10^. Long arrows indicate rapid differentiation with large gene expression changes, while short arrows represent terminally differentiated cells maintaining homeostasis^11^. Pseudotime analysis for single-cell trajectory was constructed by Monocle (version: 2.10.1). Pseudotime analysis^12^ can conceptualize the cellular differentiation and development as continuous processes.

**Statistical analysis**

Data were compared for statistical significance using GraphPad Prism version 5.01 (Graph Pad Software, San Diego, CA, USA). Student’s *t* test was used for statistical analyses. The data are presented as the mean ± SEM, and differences were considered statistically significant at **p* < 0.05, ***p* < 0.01, and ****p* < 0.001.

**REFERENCE**

1. Li H, Durbin R. Fast and accurate long-read alignment with Burrows-Wheeler transform. Bioinformatics. 2010 Mar 1;26(5):589-95.

2. Van der Auwera GA, Carneiro MO, et al. From FastQ data to high confidence variant calls: the Genome Analysis Toolkit best practices pipeline. Curr Protoc Bioinformatics. 2013;43(1110):11.10.1-11.10.33.

3. Wang K, Li M, Hakonarson H. ANNOVAR: functional annotation of genetic variants from high-throughput sequencing data. Nucleic Acids Res. 2010 Sep;38(16):e164.

4. Kumar P, Henikoff S, Ng PC. Predicting the effects of coding non-synonymous variants on protein function using the SIFT algorithm. Nat Protoc. 2009;4(7):1073-81.

5. Adzhubei IA, Schmidt S, Peshkin L, et al. A method and server for predicting damaging missense mutations. Nat Methods. 2010 Apr;7(4):248-9.

6. Schwarz JM, Cooper DN, Schuelke M, Seelow D. MutationTaster2: mutation prediction for the deep-sequencing age. Nat Methods. 2014 Apr;11(4):361-2.

7. Ernst C, Eling N, Martinez-Jimenez CP, Marioni JC, Odom DT. Staged developmental mapping and X chromosome transcriptional dynamics during mouse spermatogenesis. Nat Commun. 2019 Mar 19;10(1):1251.

8. Baker MA, Hetherington L, Weinberg A, Velkov T. Phosphopeptide analysis of rodent epididymal spermatozoa. Journal of visualized experiments : JoVE 2014; (94).

9. Kanehisa M, Araki M, Goto S, et al. KEGG for linking genomes to life and the environment. Nucleic Acids Res. 2008 Jan;36(Database issue):D480-4.

10. La Manno G, Soldatov R, Zeisel A, et al. RNA velocity of single cells. Nature. 2018 Aug;560(7719):494-498.

11. Svensson V, Pachter L. RNA Velocity: Molecular Kinetics from Single-Cell RNA-Seq. Mol Cell. 2018 Oct 4;72(1):7-9.

12. Trapnell C, Cacchiarelli D, Grimsby J, et al. The dynamics and regulators of cell fate decisions are revealed by pseudotemporal ordering of single cells[J]. Nature biotechnology, 2014, 32(4): 381.

**Supplementary Figures and Tables**

**Fig. S1** The mRNA expression of six candidate genes in mouse testicular single cell transcriptome database.

**Fig. S2** *ZNF282* compound heterozygous variants in the NOA pedigree.

**Fig. S3** *Zfp282-*KO male mice were fertile.

**Fig. S4** Transmission electron microscopy of the manchette structure in WT and *Akap4*^R428H^ mice spermatids.

**Fig. S5** Re-analysis of *Akap4* knock out mice testes single-cell RNA sequencing Data (SRA: SRR9107534).

**Fig. S6** Germ cell developmental trajectory analysis provided novel findings compared with the results from the original literature.

**Fig. S7** Enrichment analysis of round spermatids differentially expressed genes .

**Table S1** Candidate rare variants identified in NOA pedigree.

**Table S2** Primers for Sanger sequencing of *AKAP4* and *ZNF282* variants.

**Table S3** Primers of RT-PCR of *Zfp282*-KO mice.

**Table S4** Primers for genotyping of *Zfp282*-KO mice.

**Table S5** Targeting strategy to generate *Akap4*^R428H^ knock in mice.

**Table S6** BLAST of human AKAP4 and mouse AKAP4.

**
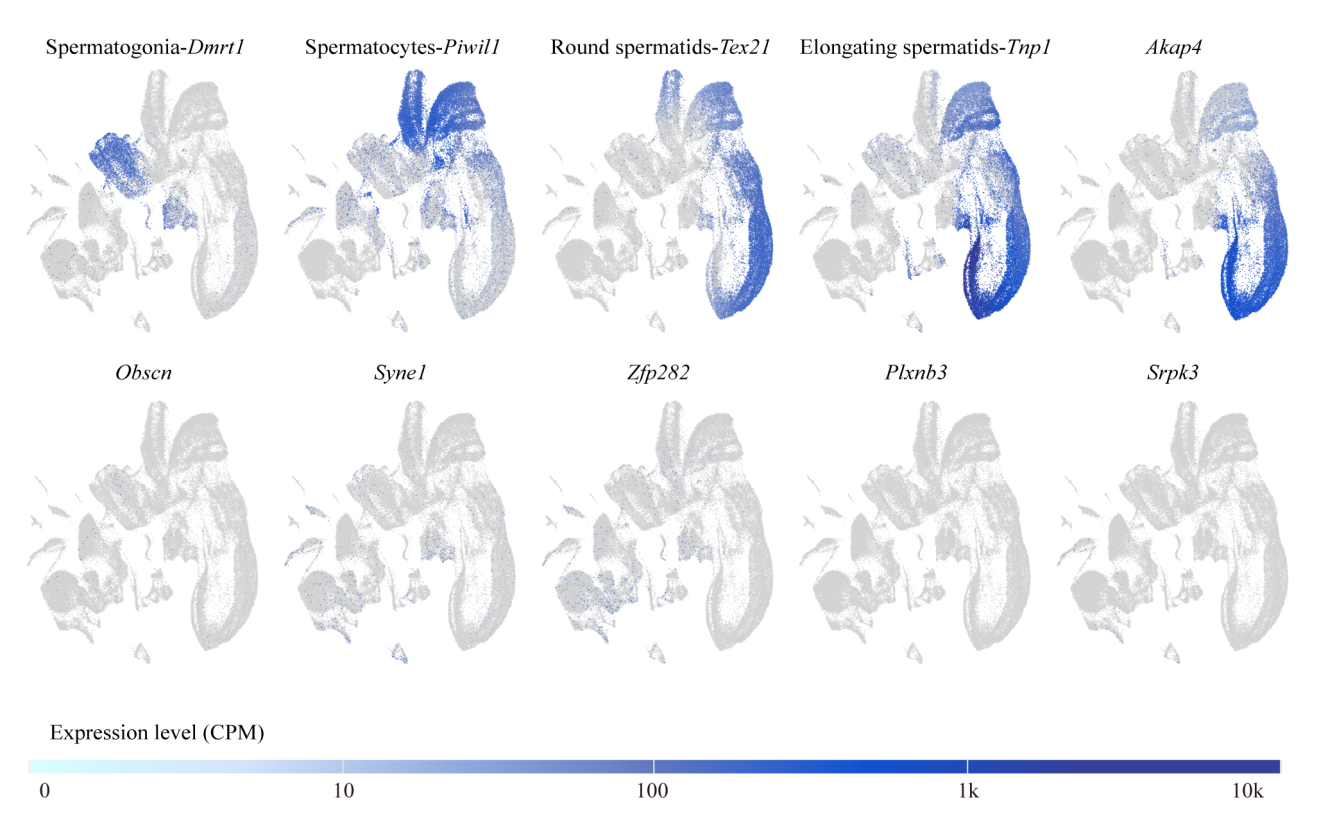
Fig. S1 The mRNA expression of six candidate genes in mouse testicular single cell transcriptome database.** Graphs were exported from online database, Single Cell Expression Atlas (https://www.ebi.ac.uk/gxa/sc/). Each dot represents a cell and the blue dot indicates that the cell expresses the target gene.

**
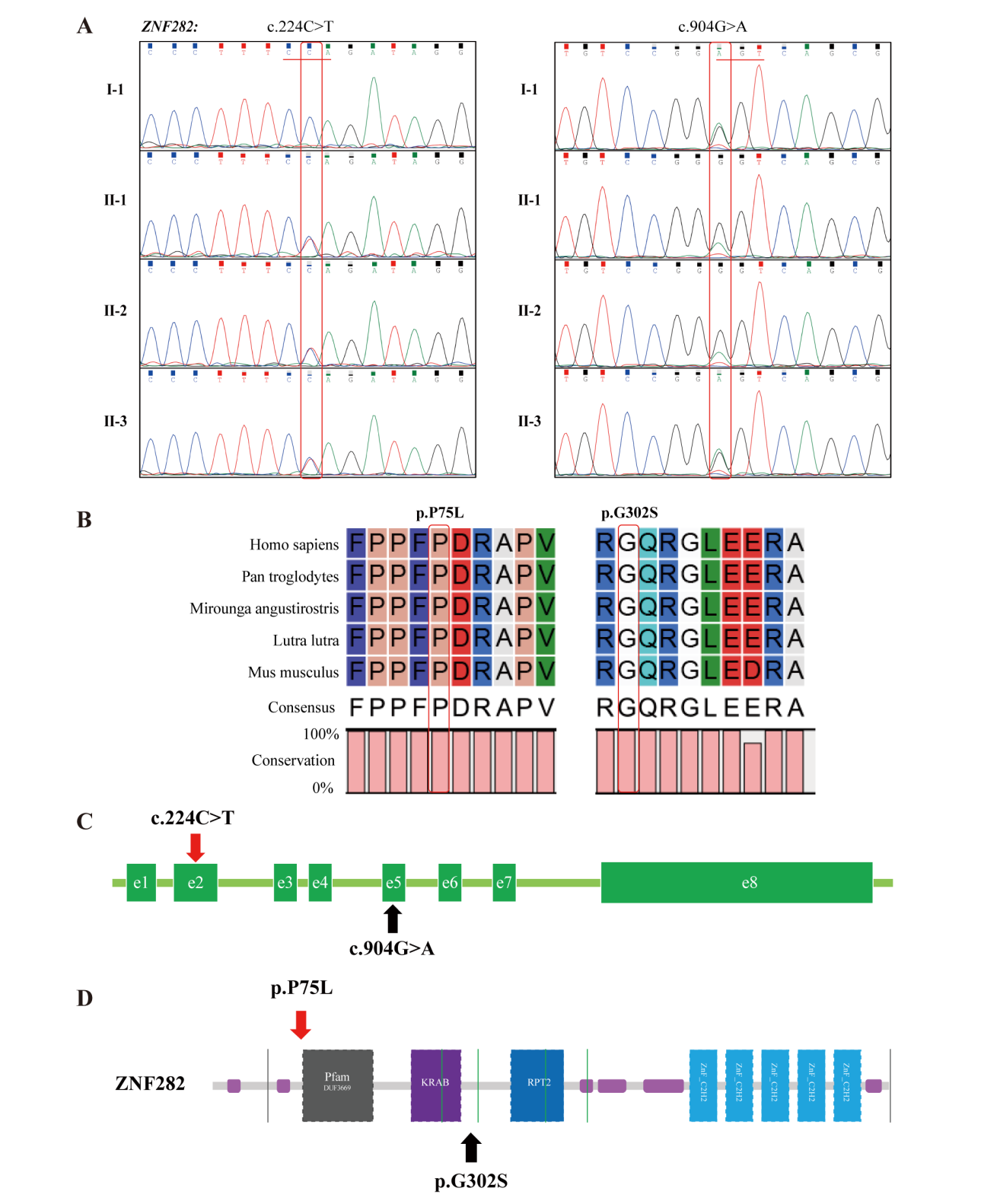
Fig. S2 *ZNF282* compound heterozygous variants in the NOA pedigree.** (**A**) Sanger sequencing of pedigree members to validate the *ZNF282* candidate variant genotype. (**B**) Conservation analysis of ZNF282 P75 and G302 amino acid. (**C**) The two *ZNF282* candidate mutations locate in exon 2 and exon 5 of *ZNF282* gene. (**D**) The location of two ZNF282 mutation amino acids.


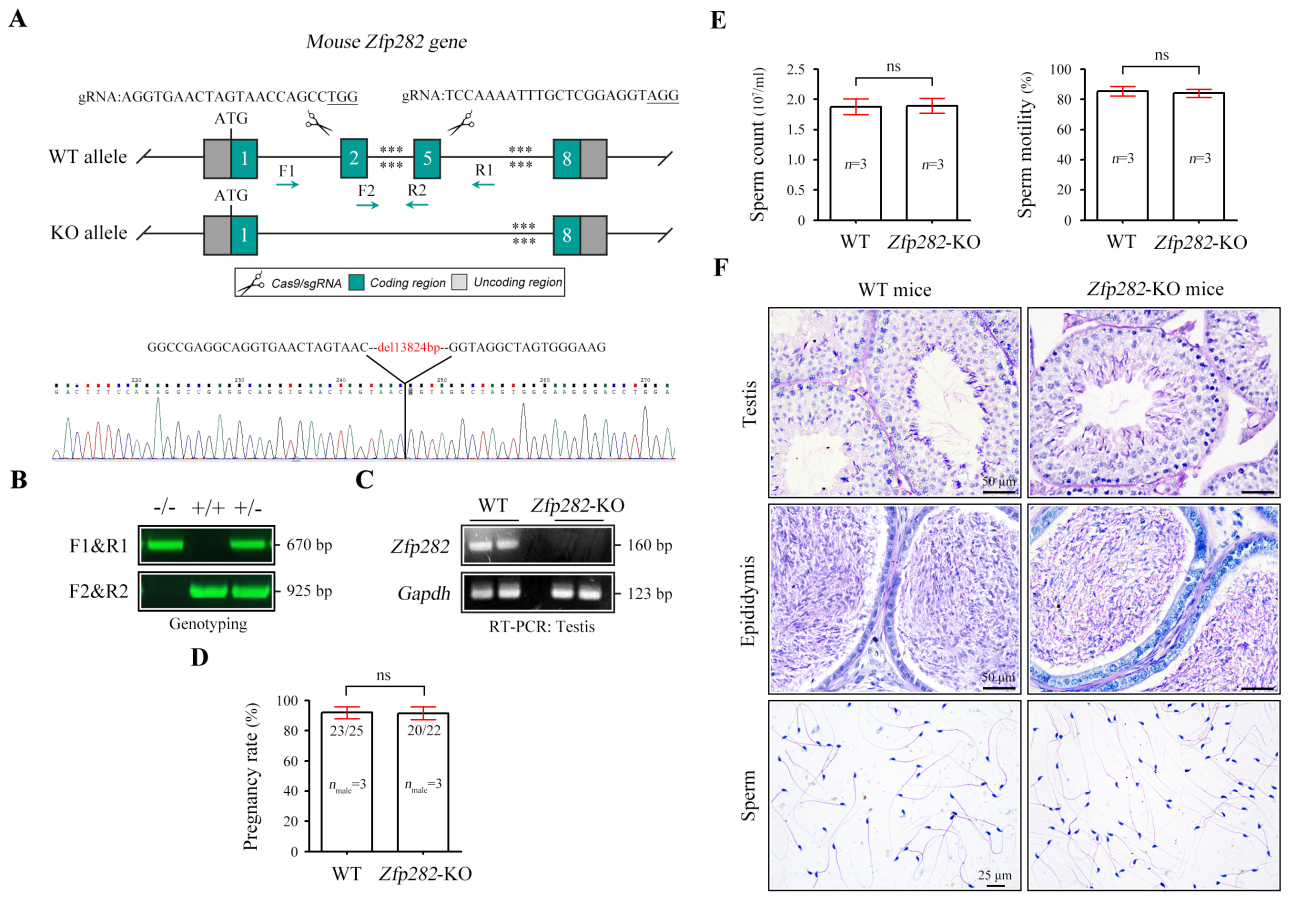
**Fig. S3 *Zfp282*-KO male mice were fertile.** (**A**) Schematic illustration of the targeting strategy for generating *Zfp282*-KO mice (mouse ZFP282 is orthologous to human ZNF282). Two gRNAs were used to target exons 2~5. (**B**) Genotyping PCR was performed using mouse tails. (**C**) RT-PCR of *Zfp282* was performed using testis mRNA of *Zfp282*-KO mice and their littermate WT mice. *Gapdh* served as a loading control. (**D**) Pregnancy rate of *Zfp282*-KO male mice and their littermate WT male mice. 23 of 25 female mice mated with WT male mice were pregnant. 20 of 22 female mice mated with *Zfp282*-KO male mice were pregnant. Data were presented as the mean ± SEM (n=3 males each group). (**E**) Sperm count and total sperm motility in *Zfp282*-KO mice and their littermate WT mice. Data were presented as the mean ± SEM (n=3 each group). Statistical significance was determined by two-tailed, unpaired Student’s t test; ns, not significance. (**F**) Representative hematoxylin-eosin staining of testis and epididymis, as well as papanicolaou staining of sperm from *Zfp28*2-KO mice and their littermate WT mice. Scale bars, 50 or 25 μm.

**
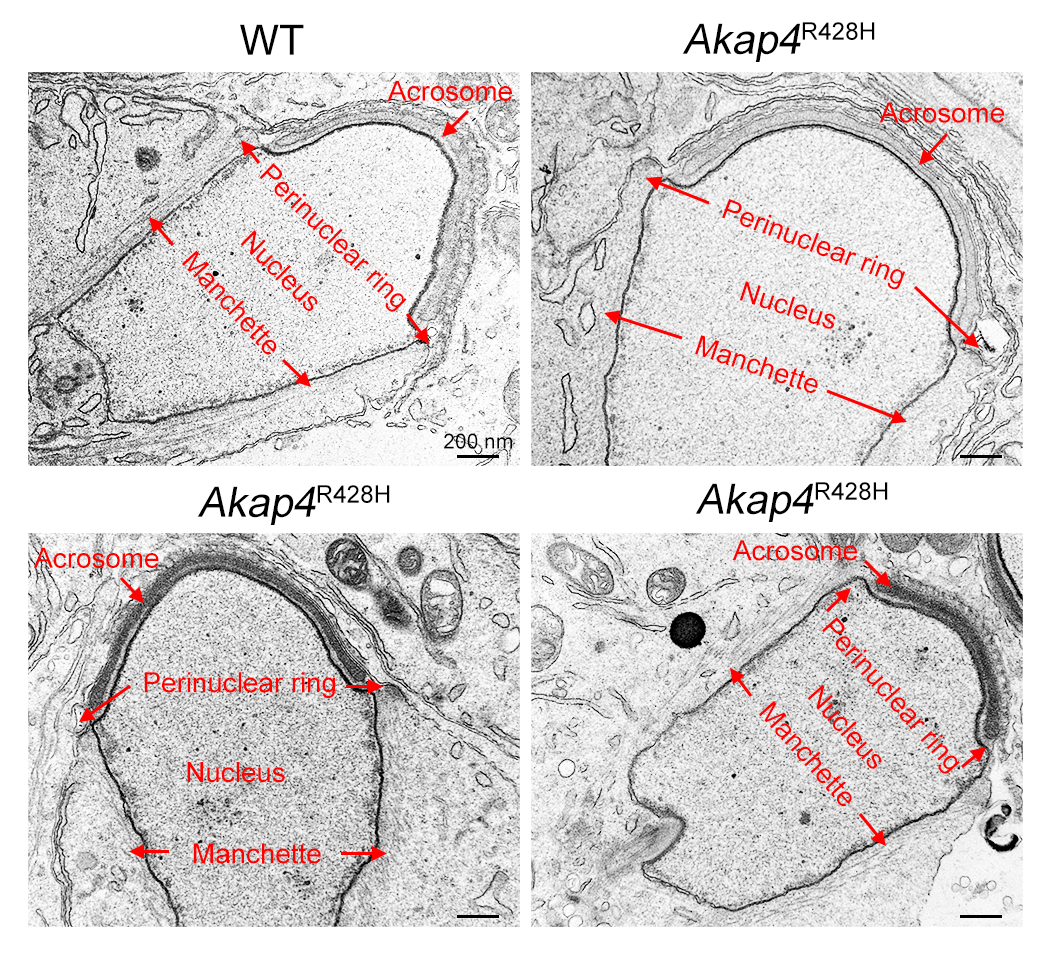
Fig. S4. Transmission electron microscopy of the manchette structure in WT and *Akap4*^R428H^ mice spermatids.** Testis seminiferous tubules from WT mice and *Akap4*^R428H^ mice were fixed and sectioned for transmission electron microscopy.


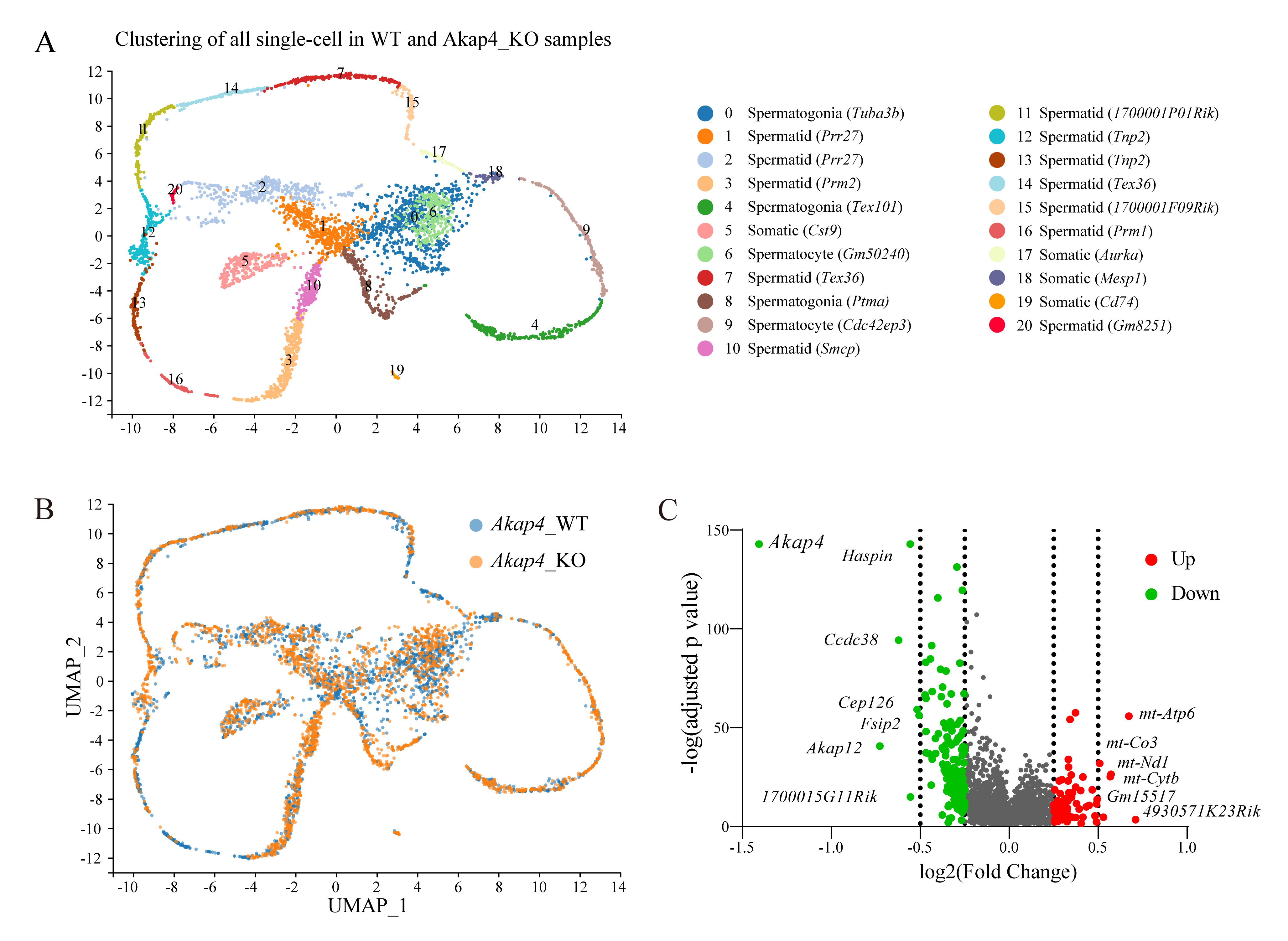
**Fig. S5 Re-analysis of Akap4 knock out mice testes single-cell RNA sequencing Data (SRA: SRR9107534).** (**A**) Testicular cells from KO mouse (n=1) and wild-type (WT) control (n=1) were subjected to clustering and annotated with maker genes (see Supplementary Materials and Methods) and the most characteristic gene of each cluster was indicated in parentheses. (**B**) Cells from WT and KO mice were visualized by different group, where each point represented a single cell and different colors indicated two distinctive samples. (**C**) Volcano plot of differential gene analysis of total testicular cell transcriptomes between *Akap4*-KO and WT mice by bulk RNA-seq analysis (|log2FC |≥ 0.5, adjusted p value <0.05).


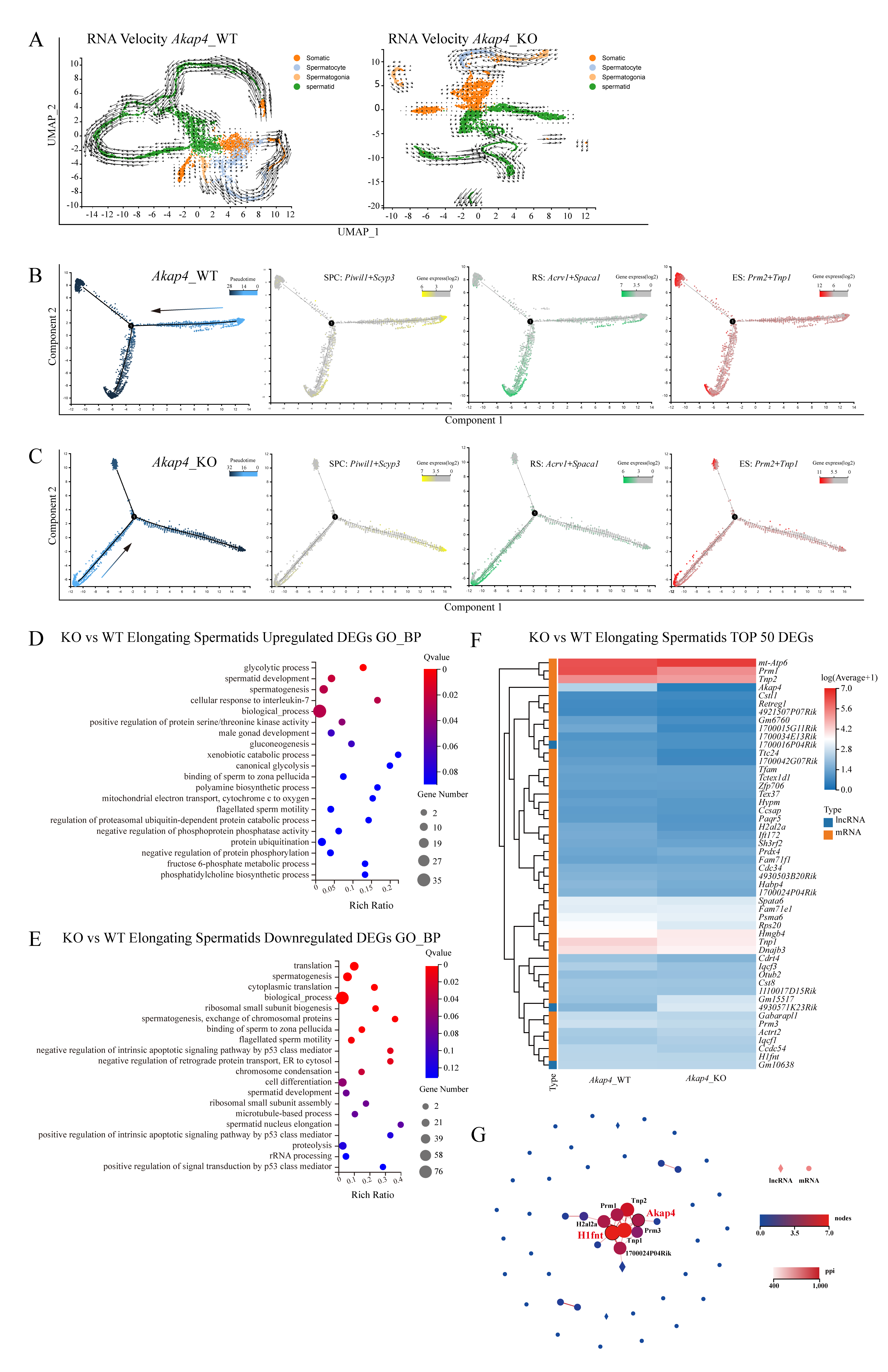


**Fig. S6 Germ cell developmental trajectory analysis provided novel findings compared with the results from the original literature. (A)** RNA velocity analysis of WT (left) and KO (right) mouse testicular germ cells. RNA velocity vectors were projected on different germ cells. Cells were colored by cell clusters. Direction of the arrows indicated the extrapolated future states of cells and length of the arrows reflected how fast the cells were heading toward a particular fate. **(B)** Pseudotime trajectory analysis of WT testicular germ cells. Trajectory analysis displayed the starting point in pseudotime with arrows. Trajectory mapping of these cells reveals a simple path with ordered progression from spermatocytes to haploid spermatids. Spermatocytes (SPC) were indicated in yellow spots with marker genes (*Piwil1* and *Sycp3*), Round spermatids (RS) were indicated in green spots with marker genes (*Arcv1* and *Spaca1*), Elongating spermatids (ES) were indicated in red spots with marker genes (*Prm2* and *Tnp1*). Each point corresponded to a single cell. (C) Pseudotime trajectory analysis of KO testicular germ cells. **(D-E)** Gene ontology_Biological Process (GO_BP) analysis of Elongating spermatids upregulated/downregulated differentially expressed genes (DEGs) (|log2FC |≥ 0.5, adjusted p value <0.05) including 295 downregulated and 59 upregulated DEGs. **(F)** Heatmap showing the expression of the Top 50 DEGs, including Top 40 downregulated DEGs and Top 10 upregulated DEGs of Elongating spermatids between *Akap4*-KO and WT mouse according to the range of log2FC value. (**J**) Protein protein interaction network (PPI) analysis of the main 50 DEGs (corresponding to Fig. S5F) showed potential AKAP4 interacting partners.

**
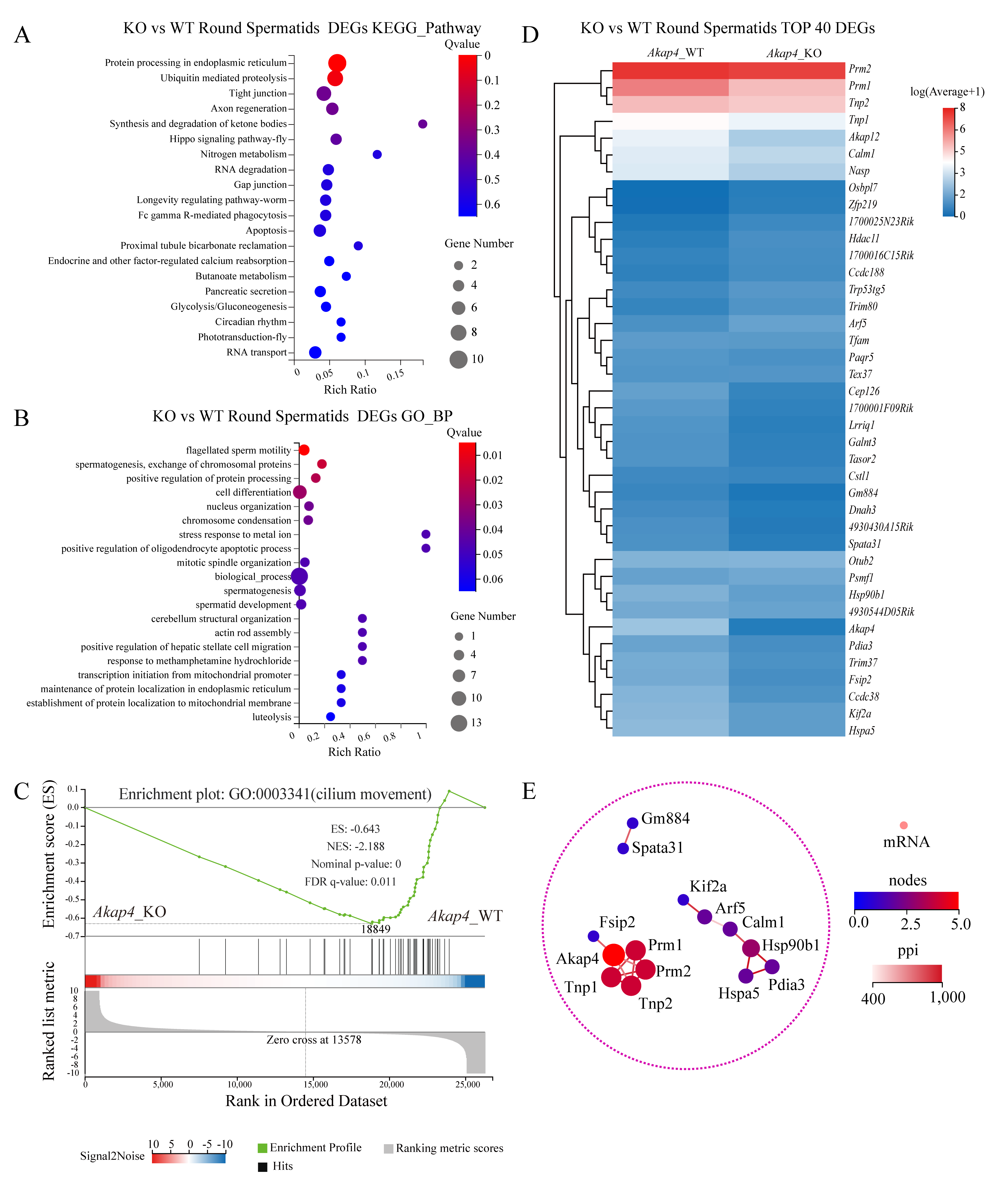
Fig. S7 Enrichment analysis of round spermatids differentially expressed genes .** **(A)** KEGG pathway analysis of round spermatids differently expressed genes (DEGs) between *Akap4*-KO and WT mouse. **(B)** Gene ontology_Biological Process (GO_BP) analysis of round spermatids DEGs (|log2FC |≥ 0.5, adjusted p value <0.05) including 46 downregulated and 50 upregulated DEGs. (**C**) Gene Set Enrichment Analysis (GSEA) was applied to the round spermatids DEGs and indicated a significant down regulation of the cilia motility pathway in the KO mouse. (**D**) Heatmap showing the Top 40 DEGs of Round spermatids between *Akap4*-KO and WT mouse including Top 30 downregulated DEGs and Top 10 upregulated DEGs of Round spermatids between Akap4-KO and WT mouse according to the range of log2FC value. **(E)** Protein protein interaction network (PPI) analysis of the main 40 DEGs (corresponding to Fig. S6D) showed potential Akap4 interacting partners.

| GENE | Genomic (hg38) | Transcripts | cDNAchange | AAchange | In silico prediction | | | | Conservation | |
| --- | --- | --- | --- | --- | --- | --- | --- | --- | --- | --- |
|  |  |  |  |  | SIFT | MutationTaste | PolyPhen2 | CADD | GERP++ | SiPhy |
| *OBSCN* | chr1 | NM_001271223.2 | c.13765G>A | p.Val4589Met | / | Polymorphism | / | 0.801 | Nonconserved | Nonconserved |
|  |  |  | c.19438G>A | p.Gly6480Ser | Tolerable | Polymorphism | Benign | 0.002 | Nonconserved | Conserved |
| *SYNE1* | chr6 | NM_182961.3 | c.20278A>G | p.Ile6760Val | Tolerable | Polymorphism | Benign | 0.892 | Nonconserved | Conserved |
|  |  |  | c.12590A>T | p.Asn4197Ile | Damaging | Polymorphism | Possibly_damaging | 22.7 | Conserved | Nonconserved |
| *ZNF282* | chr7 | NM_003575.2 | c.904G>A | p.Gly302Ser | Tolerable | Polymorphism | Benign | 2.552 | Nonconserved | Nonconserved |
|  |  |  | c.224C>T | p.Pro75Leu | Damaging | Disease_causing | Possibly_damaging | 27.3 | Conserved | Conserved |
| *AKAP4* | ChrX | NM_003886.2 | c.1286G>A | p.Arg429His | Damaging | Disease_causing | Possibly_damaging | 24.3 | Conserved | Conserved |
|  |  |  |  |  |  |  |  |  |  |  |
| *PLXNB3* | ChrX | NM_001163257.1 | c.3824T>C | p.Val1275Ala | Tolerable | Polymorphism | Benign | 0.571 | Conserved | Nonconserved |
|  |  |  |  |  |  |  |  |  |  |  |
| *SRPK3* | ChrX | NM_014370.3 | c.839G>A | p.Arg280Gln | Tolerable | Disease_causing | Benign | 23.1 | Conserved | Nonconserved |
|  |  |  |  |  |  |  |  |  |  |  |

**Table S1. Candidate rare variants identified in NOA pedigree.**

**Table S2. Primers for sanger sequencing.**

| Primers | Sequence (5’→3’) |
| --- | --- |
| *AKAP4*-1F | CTCCAGCCAAACCTCCTA |
| *AKAP4*-1R | CACCGACTTTCTCAGCACTA |
| *ZNF282*-1F | TGGCAGAAGAAAGACGACA |
| *ZNF282*-1R | AGGCTCCCAGACAGAAATG |
| *ZNF282*-2F | CCAATAGGAACCCGAGAC |
| *ZNF282*-2R | CAGAGGTAGCAGCCCATAA |

**Table S3: Primers of RT-PCR.**

| Gene | Primers | Sequence (5’→3’) | Amplicon size |
| --- | --- | --- | --- |
| *Zfp282* | Forward | TCGCTTTACCTGGGAGGATATGT | 160 bp |
|  | Reverse | ACATATCCTCCCAGGTAAAGCGA |  |
| *Gapdh* | Forward | AGGTCGGTGTGAACGGATTTG | 123 bp |
|  | Reverse | TGTAGACCATGTAGTTGAGGTCA |  |

**Table S4: Primers for genotyping of *Zfp282*-KO mice.**

| **PCR No.** | **Primer No.** | **Sequence (5’→3’)** | **Band size** |
| --- | --- | --- | --- |
| PCR (1) | F1 | GCTGCTTCTTTGTTCCTCAATTTC | KO: 670 bp |
|  | R1 | CCCATTTGACACCAGTGAAGGC |  |
| PCR (2) | F2 | AAAGGAAGAGCGATGGATGCAG | WT: 925 bp |
|  | R2 | CCCATTTGACACCAGTGAAGGC |  |

**Table S5 Targeting strategy to generate *Akap4*^R428H^ knock in mice.**

| **Items** | **Contents** | **Off-target analysis** |
| --- | --- | --- |
| gRNA-A1 (matches forward strand of gene) | GGAGGCCATGCTAAAGCGTC**TGG** | 0.65 |
| gRNA-B1 (matches reverse strand of gene) | ACTGACCAGACGCTTTAGCA**TGG** | 0.71 |
|  | Legend for the CRISPRater score: LOW efficacy (score<0.56); MEDIUM efficacy (0.56≤score<0.74); HIGH efficacy (score≥0.74). | |
| Donor oligo sequence | GAGGAATCTTTTCAATCATGGTAAACAAAATGCAGCGGACATCATGGAGGCCATGCTAAAACATCTGGTCAGTGCTCTTCTTGGTGAGAAGAAGGAGACTAAGTCTCAAAGTCTGGCCTATGC  (Note: The mutation sequence is colored in red, synonymous mutation sequences is colored in blue.) | |
| Primer for Genotyping |  | |
| Mouse *Akap4*-F | GACATGATGGTCTCTGTTATGA | |
| Mouse *Akap4*-R | CTCTCTTCTCATCTTTACTGGTG | |
| PCR product size | 574 bp | |

**Table S6 BLAST of human AKAP4 and mouse Akap4.** The R428H mutation we are creating in the mouse *Akap4* gene is equivalent to the R429H mutation in the human *AKAP4* gene.


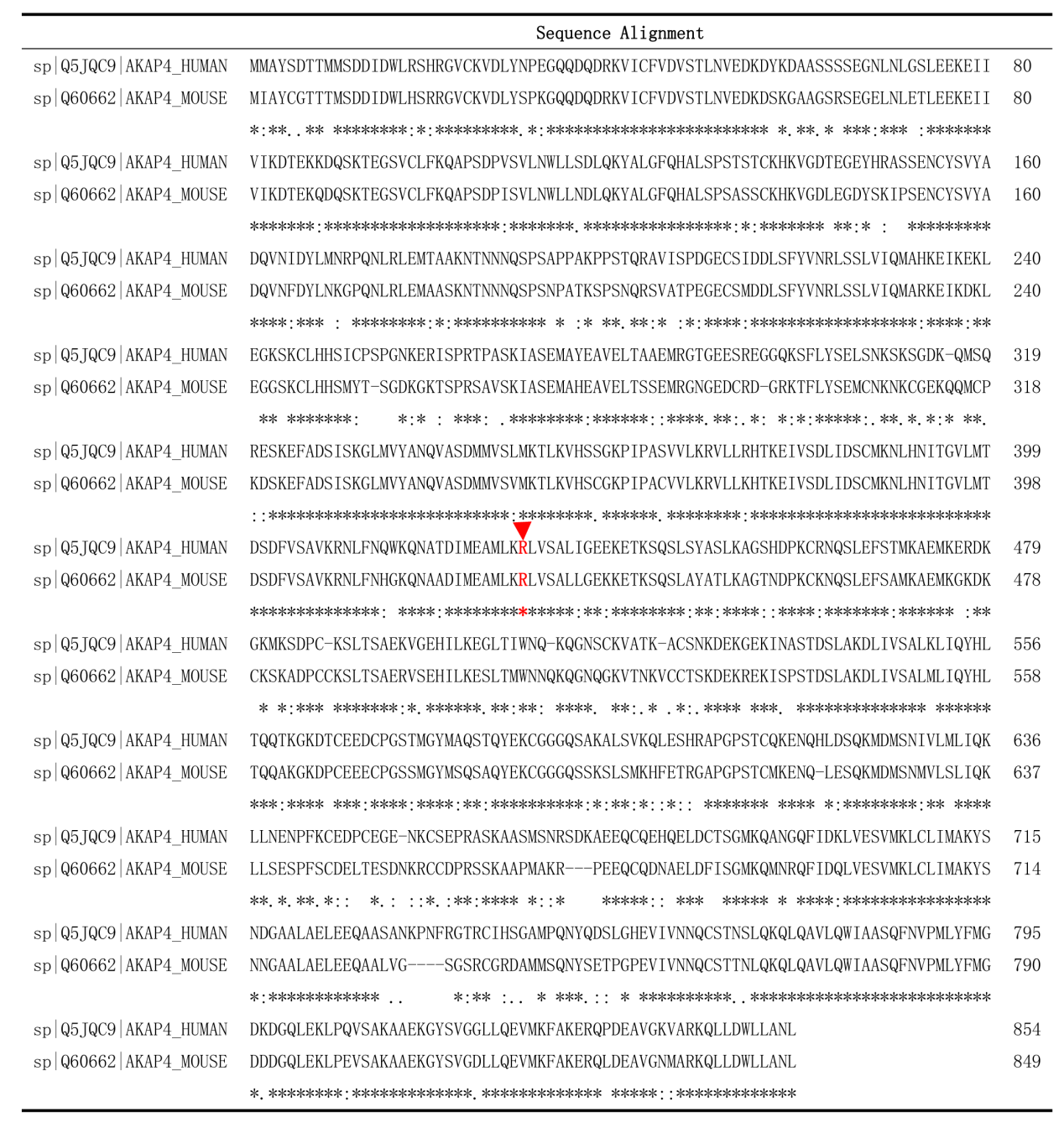

Supplement: Supplementary file 1 — Supporting information [file CTM2-13-e1463-s001.docx]
